# Melatonin Attenuates Androgen-Induced Visceral Adiposity in an Experimental Polycystic Ovary Syndrome Model

**DOI:** 10.7759/cureus.100024

**Published:** 2025-12-24

**Authors:** Nina Tirso, Emina Kiseljakovic, Almir Fajkić, Samra Custovic, Maida Sahinovic, Orhan Lepara

**Affiliations:** 1 Department of Obstetrics and Gynaecology, General Hospital, Sarajevo, BIH; 2 Department of Medical Biochemistry, University of Sarajevo - Faculty of Medicine, Sarajevo, BIH; 3 Department of Pathophysiology, University of Sarajevo - Faculty of Medicine, Sarajevo, BIH; 4 Department of Histology and Embryology, University of Sarajevo - Faculty of Medicine, Sarajevo, BIH; 5 Department of Human Physiology, University of Sarajevo - Faculty of Medicine, Sarajevo, BIH

**Keywords:** antioxidants, melatonin, metformin, pcos, rat model, testosterone, visceral fat

## Abstract

Introduction: Polycystic ovary syndrome (PCOS) represents a state of androgen-driven metabolic dysregulation where visceral adiposity and inflammation critically define cardiometabolic risk. Visceral adiposity is not a bystander in PCOS; it is an active endocrine organ driving insulin resistance, low-grade inflammation, and androgen persistence. Interventions that reverse adipocyte hypertrophy and inflammatory signaling may therefore alter the metabolic trajectory of PCOS. Beyond its chronobiotic role, melatonin exerts profound metabolic actions via MT1/MT2 receptors in adipose tissue, modulating oxidative stress and inflammatory gene expression. Yet its direct impact on androgen-induced visceral adiposity remains unclear.

Aim: The present study aimed to evaluate the effects of melatonin, metformin, and their combination on visceral fat accumulation in a testosterone-induced PCOS rat model.

Material and methods: Thirty prepubertal female Wistar rats were randomized into five groups (n=6): control, PCOS (testosterone 20 mg/kg/day), PCOS+metformin (500 mg/kg/day), PCOS+melatonin (2 mg/kg/day), and PCOS+melatonin+metformin. Treatments lasted 36 days. Estrous cyclicity was monitored by daily vaginal cytology, and somatometric parameters were recorded weekly. On day 36, serum, ovaries, and visceral fat were collected for biochemical and histological analysis.

Results: Vaginal smear changes and ovarian pathological alteration due to prolonged testosterone exposure confirmed the successful induction of the PCOS model. Measures of central adiposity, including abdominal circumference and the TC/AC ratio, were significantly higher in the PCOS model than in controls (p < 0.001). Abdominal circumference (AC) increase was greatest in the PCOS model (p < 0.001), while all treatment groups showed significant reductions, most notably in the melatonin + metformin group, followed by melatonin monotherapy and then metformin (all p < 0.001 vs. PCOS). Melatonin was more effective than metformin (p=0.029). AC/TC reduction was greatest in the combined treatment group (p < 0.05). Total weight gain among groups did not reach statistical significance.

While total visceral fat weight did not differ among groups, histology revealed a marked reduction in adipocyte number in treated animals, most pronounced in the melatonin group (p < 0.033).

Conclusion: Our findings identify melatonin as a metabolic modulator of androgen-driven adiposity, supporting its potential as an adjunctive therapy targeting visceral fat and inflammation in PCOS

## Introduction

Polycystic ovary syndrome (PCOS) is the most common endocrine and metabolic disorder in women, affecting 10-13% of those of reproductive age worldwide [1,2]. In adolescents aged 10-24 years, its prevalence ranges from 6-18% [3]. The syndrome manifests through a triad of elevated androgens, disrupted folliculogenesis, and anovulation [4]. Despite decades of investigation, its origin remains elusive, arising from a complex interplay of genetic predisposition, environmental factors, and neuroendocrine-metabolic crosstalk. Among its many features, hyperandrogenism (HA) is the most consistent, present in over three-quarters of cases [5], and excessive ovarian androgen production is now regarded as a central driver of its pathophysiology [6].

PCOS extends far beyond reproductive dysfunction. It represents a systemic metabolic disorder shaped by dysregulation of the hypothalamic-pituitary-ovarian axis, insulin resistance (IR), compensatory hyperinsulinemia, oxidative stress (OS), and chronic low-grade inflammation (LGI). Yet, the directionality of these disturbances remains unclear: whether hyperandrogenism initiates metabolic derangement or whether inflammation and insulin resistance amplify androgen excess continues to be debated. Clinically, women with PCOS often exhibit IR, dyslipidemia, and increased risk of type 2 diabetes, features that redefine PCOS as a disorder with profound cardiometabolic implications rather than one confined to infertility or menstrual irregularity [7,8].

Abdominal obesity is reported in 60-70% of women with PCOS and contributes to their increased risk of cardiovascular disease, malignancy, and pregnancy complications. Strikingly, this pattern is not confined to the obese phenotype; it is also seen in a substantial proportion of overweight and even normal-weight women. The interdependence between androgen excess and abdominal adiposity forms a self-reinforcing loop: androgens favor central fat deposition, while visceral fat perpetuates androgen production through autocrine, paracrine, and endocrine mechanisms, as well as indirectly via IR and hyperinsulinemia [9].

Visceral adipose tissue acts as an active endocrine organ rather than a passive storage site. It secretes adipokines and pro-inflammatory cytokines such as adiponectin, leptin, resistin, C-reactive protein (CRP), IL-1β, IL-6, IL-18, and TNF-α, which further disturb lipid and glucose metabolism [10]. As adipocytes enlarge, regional hypoperfusion and hypoxia activate the nuclear factor kappa-light-chain-enhancer of activated B cells (NF-κB) pathway, promoting transcription of inflammatory genes and recruitment of macrophages. This cascade establishes a chronic inflammatory milieu that impairs insulin sensitivity and accelerates metabolic deterioration [11]. Despite extensive research, the specific contribution of individual cytokines to PCOS pathogenesis remains unresolved [12].

Inflammatory markers, including CRP, IL (1,6,8,17), TNF-α, the neutrophil-to-lymphocyte ratio (NLR), and platelet-to-lymphocyte ratio (PLR), have been explored as accessible surrogates of systemic inflammation in PCOS [13,14]. Among them, the CRP/albumin ratio shows superior diagnostic performance, independent of body mass index (BMI), compared with free androgen or IR indices. NLR, conversely, has been linked with poor cardiovascular health and metabolic syndrome (MS) and may downregulate follicle-stimulating hormone (FSH), indirectly sustaining androgen production. The role of obesity in this inflammatory landscape remains controversial: while it clearly amplifies both OS and LGI, the metabolic burden in lean PCOS phenotypes underscores that these disturbances are not merely weight-driven [15,16].

Visceral adiposity is typically assessed by waist circumference (WC), waist-to-hip ratio (WHR), or imaging modalities [17]. Reducing visceral fat and suppressing inflammation represent two converging therapeutic targets. Pharmacological approaches such as metformin or glucagon-like peptide-1 (GLP-1) receptor agonists have shown efficacy in this regard, while lifestyle interventions (caloric restriction, structured exercise, and stress reduction) remain essential [18]. Anti-inflammatory interventions have also demonstrated favorable metabolic effects in PCOS [19].

Melatonin, a pineal indoleamine best known for regulating circadian rhythm, has emerged as an intriguing adjunct in PCOS management. Its metabolic influence extends beyond chronobiology: melatonin modulates lipid metabolism, supports mitochondrial function, and exerts robust antioxidant and anti-inflammatory effects. Acting through MT1 and MT2 receptors, expressed across endocrine and metabolic tissues, it also directly scavenges reactive oxygen species (ROS). In women with PCOS, melatonin concentrations are paradoxically elevated in serum and urine yet decreased in follicular fluid - suggesting compensatory systemic release in response to oxidative stress and local depletion within the ovary. Through its antioxidant capacity, melatonin protects ovarian follicles from atresia and supports ovulatory function [20,21]. Preclinical and clinical evidence indicates that melatonin supplementation improves insulin sensitivity, optimizes lipid profiles, and reduces androgen excess. Nevertheless, its direct impact on visceral adiposity and the accompanying inflammatory remodeling remains insufficiently characterized.

Given that PCOS is characterized by abdominal obesity and chronic low-grade inflammation, the study aimed to determine whether melatonin, through its antioxidant and anti-inflammatory actions, could mitigate visceral fat accumulation and related histological changes in an androgen-induced rat model of PCOS.

Animal models have been indispensable for dissecting the pathophysiology of PCOS. The rat, with its short 4-5-day estrous cycle, provides a practical framework for monitoring reproductive and metabolic fluctuations. Persistent estrus (PE), defined by at least two consecutive anovulatory cycles confirmed by cornified vaginal smears over 10 days, represents a key marker of the polycystic ovarian condition [22]. Induced PCOS in rats recapitulates the ovarian and metabolic disturbances characteristic of human PCOS [23]. Among various paradigms, testosterone-induced PCOS most closely replicates the human phenotype, encompassing HA, disrupted folliculogenesis, cycle irregularity, and metabolic alterations. The prepubertal androgen (PPA) model, in which exogenous androgens are administered during a critical developmental window, permanently remodels ovarian physiology and reproduces hallmark PCOS features, though without the typical rise in basal LH [24]. Chronic androgen exposure also promotes visceral adiposity, disrupts lipid metabolism, and activates inflammatory signaling [25].

Melatonin has thus been explored as a potent modulator of endocrine, metabolic, and reproductive features in PCOS [26]. Building on this rationale, we hypothesized that melatonin could attenuate androgen-induced visceral adiposity and histological remodeling by interfering with mitochondrial and inflammatory pathways in this model.

## Materials and methods

Animals and experimental protocols

The experiment was conducted on healthy, prepubertal female albino Wistar rats (three weeks old, with a natural body weight range of 14-44 g observed in our colony) obtained from the Animal Facility of the Faculty of Veterinary Medicine, University of Sarajevo. All animals were age-matched, clinically healthy, and underwent a two-week acclimatization period. Randomization ensured comparable distribution across groups, and dosages were adjusted weekly according to weight. Animals were maintained under standard laboratory conditions (12 h light/dark cycle, 23 ± 3 °C, 50 ± 10% relative humidity) with free access to a standard pellet diet and water ad libitum. All experimental procedures were approved by the Ethics Committee of the Faculty of Medicine, University of Sarajevo (Approval No. 02-3-4-AK-2725/24) and performed in accordance with international guidelines for the care and use of laboratory animals and the principles of the 3R rule.

After a 2-week acclimatization period, the 30 rats were randomly divided into 5 groups of 6 animals each. The control group received 1 mL of saline subcutaneously. The PCOS model group was administered testosterone propionate at a dose of 20 mg/kg/day subcutaneously. The PCOS plus metformin group received the same testosterone protocol combined with metformin at 500 mg/kg/day given orally. The PCOS plus melatonin group received testosterone along with melatonin at a dose of 2 mg/kg/day administered intraperitoneally. The final group, which received a combination of melatonin and metformin, was treated with testosterone 20 mg/kg/day subcutaneously, melatonin 2 mg/kg/day intraperitoneally, and metformin 500 mg/kg/day orally.

The treatment lasted 36 days [24]. Anthropometric measurements, including body weight, abdominal circumference, and thoracic circumference, were recorded at baseline, then weekly, and again at the end of the study to adjust treatment doses.

Estrous cycle evaluation

Vaginal cytology was performed daily throughout the experimental period to monitor estrous cyclicity. Vaginal cytology was performed daily throughout the experimental period to monitor estrous cyclicity. Smear collection began on day 14 of treatment (i.e., after 14 days of androgen or control exposure), allowing assessment of persistent estrus during the second half of the experimental protocol. The proportions of epithelial, cornified, and leukocyte cells were used to identify the cell cycle phase. Persistent estrus was defined as the predominance of cornified epithelial cells for ≥10 consecutive days, confirming successful PCOS induction.

Sample collection and processing

On day 36, rats were anesthetized with ketamine hydrochloride (80 mg/kg, i.m.) and euthanized by cardiac puncture. Blood samples were collected directly from the heart, allowed to clot, and centrifuged at 3000 rpm for 15 min. Serum was separated and stored at -80 °C until analysis. Ovaries and visceral fat depots were carefully excised, weighed, and fixed in 10% neutral buffered formalin for histological processing.

Biochemical analysis

Serum levels of tumor necrosis factor-alpha (TNF-α; Cat. No. KRC3011, Invitrogen) and interleukin-1 beta (IL-1β; Cat. No. BMS630, Invitrogen) were measured using enzyme-linked immunosorbent assay (ELISA) kits according to the manufacturer’s protocols. All assays were performed in duplicate, and absorbance was measured at 450 nm using a microplate reader.

Statistical analysis

Statistical data analysis was performed using SPSS (Statistical Package for the Social Sciences), version 13.0. The Shapiro-Wilk test was used to assess the normality of continuous variables. The results were presented as the mean (X) and standard deviation (SD), the median and interquartile interval, and absolute and relative numbers. Comparison of values was performed by one-way analysis of variance (ANOVA), after which Bonferroni’s test for multiple comparisons was applied. The significance of differences in quantitative variables that did not follow a normal distribution was tested using the Mann-Whitney test for two study groups or the Kruskal-Wallis test for three or more study groups. The degree of correlation was determined by the Pearson or Spearman method. A value of p<0.05 was considered a statistically significant difference.

## Results

**Figure 1:**
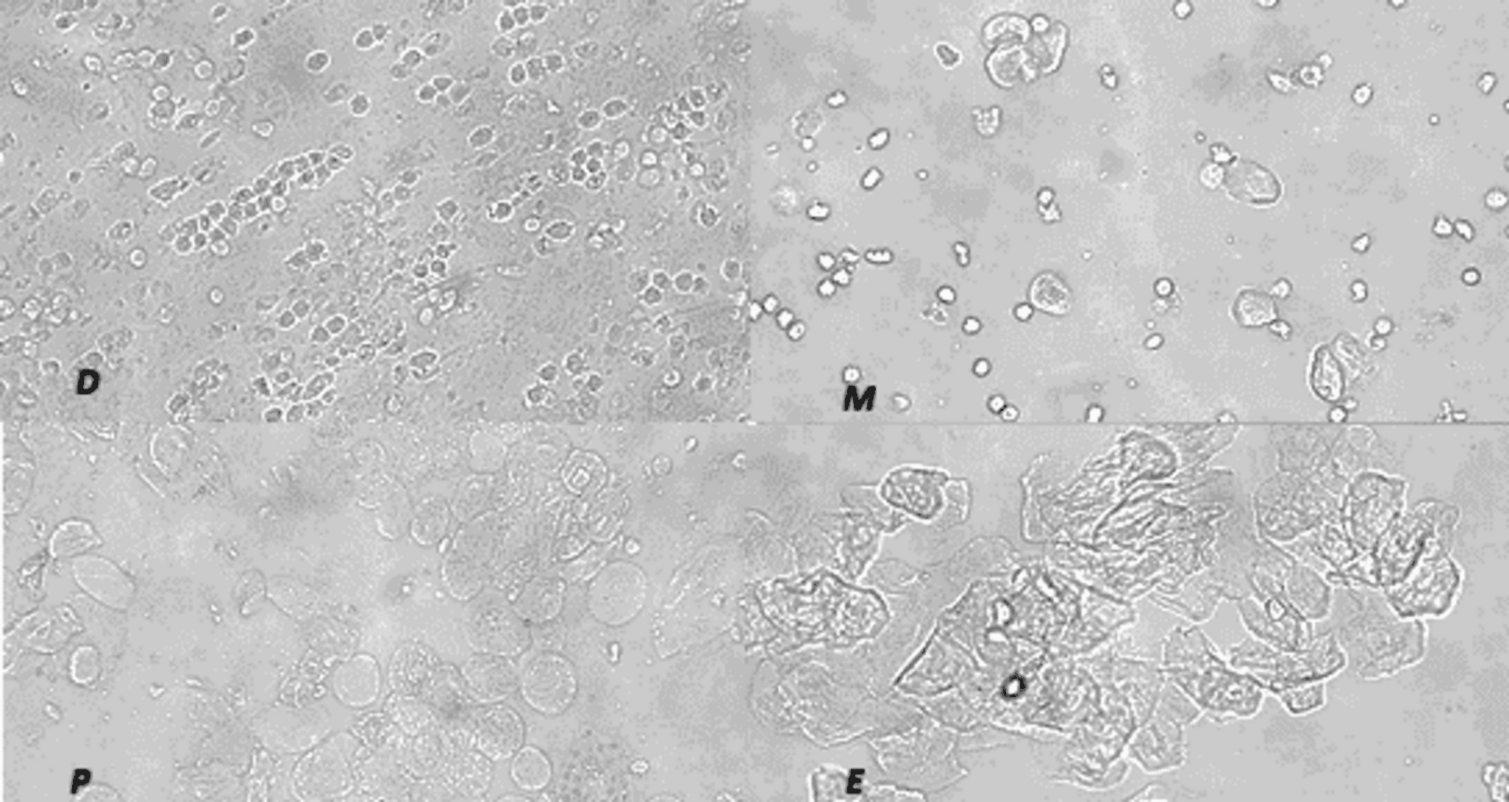
Vaginal smears: Vaginal exfoliated cells of rats were observed under a light microscope As shown in the pictures, large numbers of leukocytes and a small amount of mucus are observed in Diestrus (D). In Proestrus (P), most cells are nucleated epithelial cells. In Estrus (E), large numbers of keratin epithelial cells are observed. In Metestrus (M), several types of cells are observed, including nucleated epithelial cells, keratin epithelial cells and leukocytes. The estrous cycle of rats in the control group lasts 4- 5 days, whilst that of rats in the PCOS model group was disordered or even remained in the estrous interphase.

**Figure 2:**
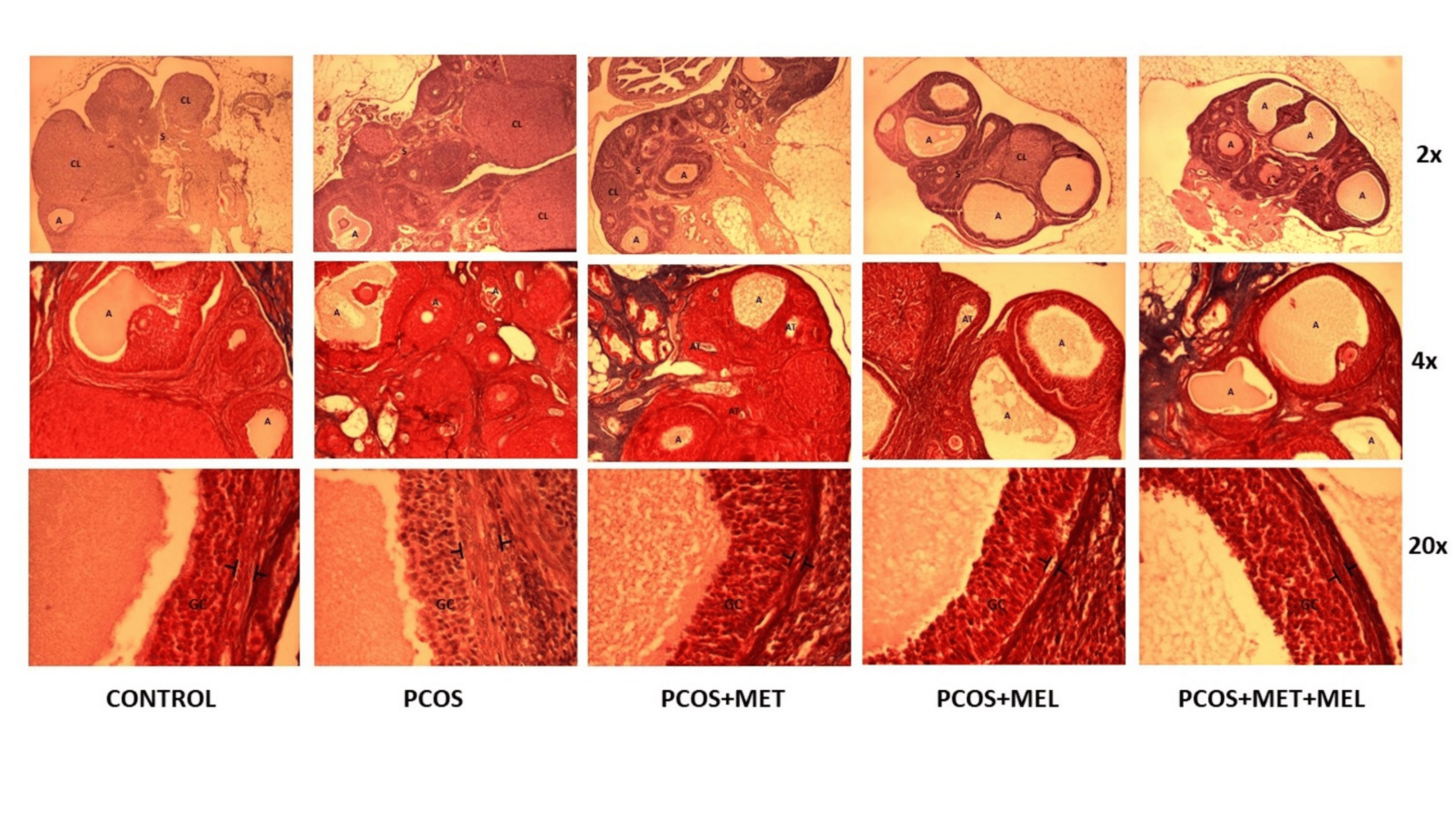
Representative photomicrographs of ovaries - HE staining (first row); Masson trichrome staining (second row); follicle wall - Masson trichrome staining (third row) GC - granulosa cells; theca interna is indicated in brackets. Pathological morphology of rat ovarian tissues stained with HE. As shown in photos, no structural abnormalities were present in the control group rats: the follicles and corpora lutea were in varying stages of development, and the granulosa cell and theca interna layers were normal. Differences were observed in the PCOS model group: the number of antral follicles increased, and theca layer was abnormally thickened, while the granulosa cell layer showed loss of lamellarity. The metformin group had a higher frequency of antral and atretic follicles, occasional small corpora lutea, and massive stroma. In the melatonin group, domination of large antral follicles and prominent stroma is observed, while theca interna appears of regular thickness. The combination therapy group had large antral follicles and numerous other developmental stages with rare corpora lutea. Granulosa and theca cells are clearly separated, and the stroma is pronounced.

The presented study showed that the PCOS model group rats were in sexual cycle disorder according to the classification of vaginal exfoliated cells (Figure 1). PCOS model group rats exhibited physical changes, including hair that was less glossy, coarser, and harder in texture. Moreover, the ovarian pathological morphology of the PCOS model group rats was greatly altered (Figure 2).

Body measurements

No significant differences in overall body weight gain were observed among groups (p = 0.752). However, other anthropometric indicators differed markedly (p < 0.001). The increase in abdominal circumference (AC) was greatest in the PCOS group (7.16 ± 0.60 cm) compared with the control (4.0 ± 0.44 cm, p < 0.001), PCOS + metformin (5.41 ± 0.73 cm, p = 0.012), PCOS + melatonin (3.83 ± 0.93 cm, p < 0.001), and combination group (3.33 ± 1.21 cm, p < 0.001). Thoracic circumference (TC) increased most in the PCOS + metformin group (5.08 ± 1.28 cm), exceeding the control (3.41 ± 0.49 cm, p = 0.035), PCOS + melatonin (3.41 ± 1.24 cm, p = 0.035), and combination group (1.91 ± 0.66 cm, p < 0.001). The TC/AC ratio also differed significantly between groups (p = 0.008), with the greatest reduction observed with the melatonin + metformin combination.

**Figure 3:**
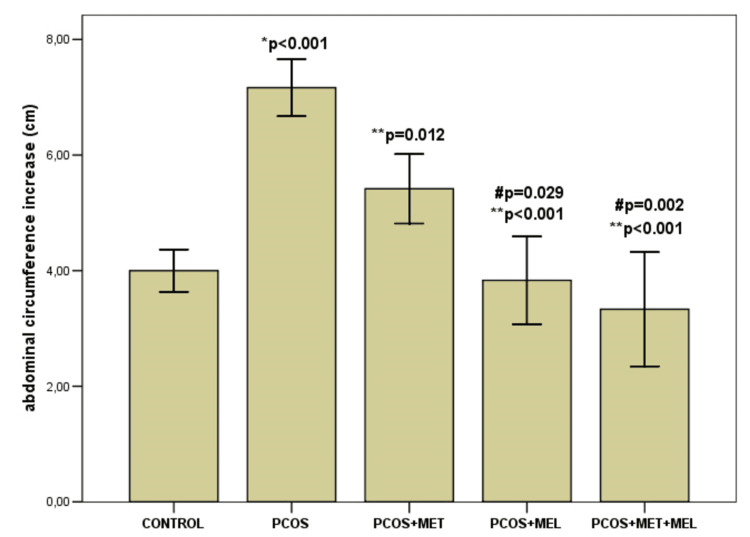
Abdominal circumference difference among groups Results are presented as mean ± standard deviation (x± SD); PCOS – model; AC-abdominal circumference; * – significant to control group; ** – significant to PCOS, # - significant to PCOS & metformin.

A modest difference in body length was noted between the PCOS and PCOS + metformin groups (p = 0.037) (Table 1, Figure 3).

**Table 1:** Anthropometric indices Comparison of values was performed by one-way analysis of variance (ANOVA), after which Bonferroni’s test for multiple comparisons was applied. Results are presented as mean ± standard deviation (x± SD), CG – control group; PCOS – model, WG – weight gain, AC-abdominal circumference; TC – thoracic circumference, L- length * – significant to CG; ** – significant to PCOS; # - significant to PCOS & metformin.

Variable	CG	PCOS	PCOS+ metformin	PCOS+ melatonin	PCOS+ melatonin+ metformin	p
WG (g)	111.66±19.11	116.33±5.27	121.33±23.95	102.00±17.15	111.0±18.12	0.752 F=0.478
AC (cm)	4.0±0.44	7.16±0.60 *p<0.001	5.41±0.73 **p=0.012	3.83±0.93 **p<0.001 ^#^p=0.029	3.33±1.21 **p<0.001 ^#^p=0.002	<0.001 F=21.128
TC (cm)	3.41±0.49	4.91±0.37	5.08±1.28 *p=0.035	3.41±1.24 ^#^p=0.035	1.91±0.66 **p<0.001 ^#^p<0.001	<0.001 F=12.578
TC/AC	0.87±0.08	0.68±0.02	0.92±0.06	0.88±0.06	0.60±0.06^#^^#^p=0.024	0.008 F=4.420
L (cm)	6.83±1.63	7.83±0.75	5.66±0.25 **p=0.037	6.16±1.50	6.50±1.14	0.043 F=2.888

Inflammatory parameters 

**Figure 4:**
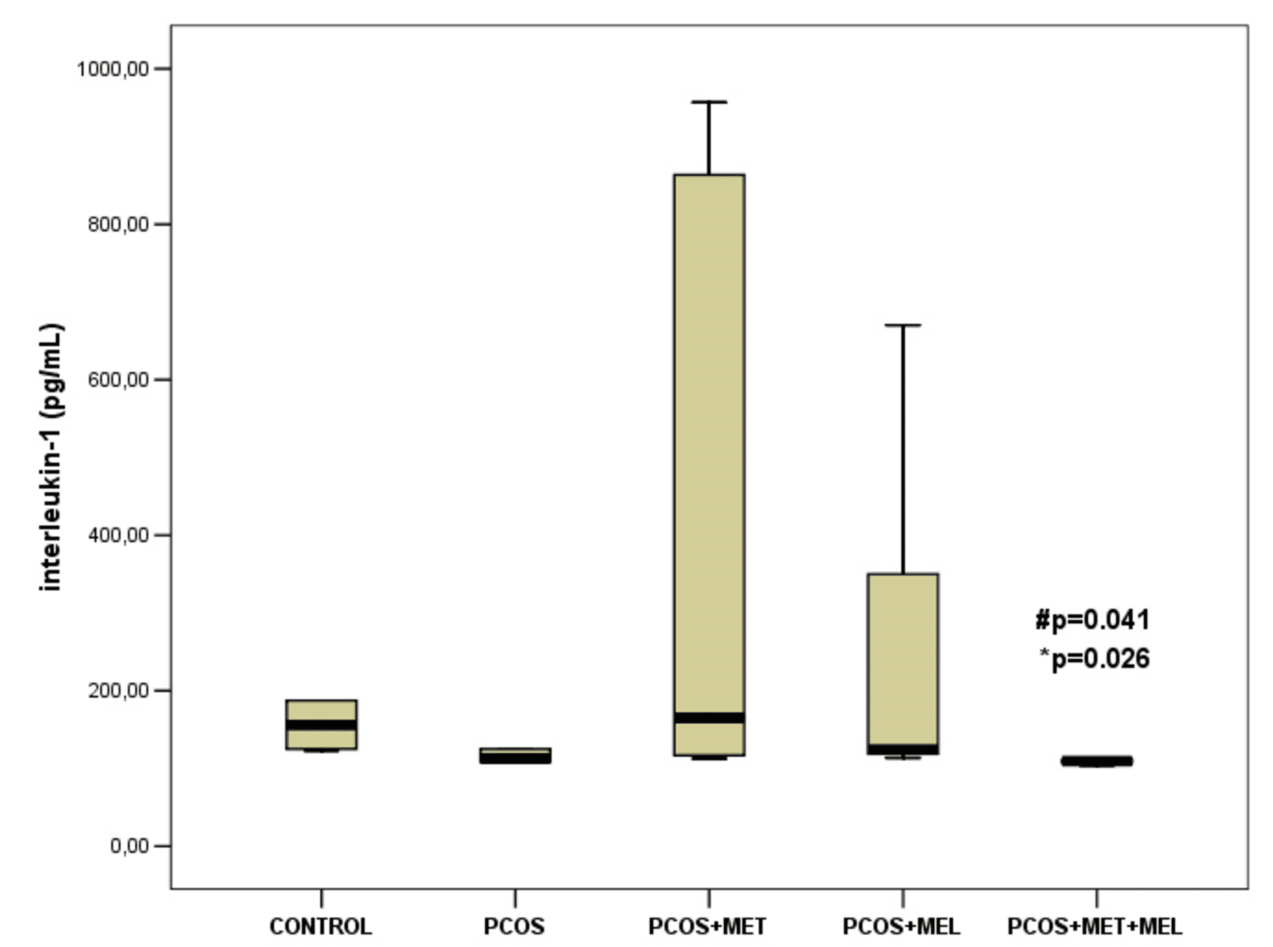
Differences in Interleukin-1β level among groups Results are shown as median (interquartile range; 25-75 percentile), IL-1β – interleukin 1 beta; * significant to PCOS&metformin, # - significant to PCOS & melatonin.

Significantly different values of interleukin-1β (IL-1β) were observed among all experimental groups (p = 0.046), whereas tumor necrosis factor-alpha (TNF-α) showed no significant variation (p > 0.05) (data not shown). The lowest IL-1β concentrations was noted in the combined therapy group - PCOS + melatonin + metformin [109.30 (106.80-210.90) pg/mL], and it was significantly lower compared to in the PCOS + metformin group [165.0 (115.75-886.87) pg/mL] (p=0,026) and PCOS + melatonin group [124.0 (114.55-431.10) pg/mL] (p=0,041) (Figure 4).

Histological analysis of visceral adipose tissue

**Figure 5:**
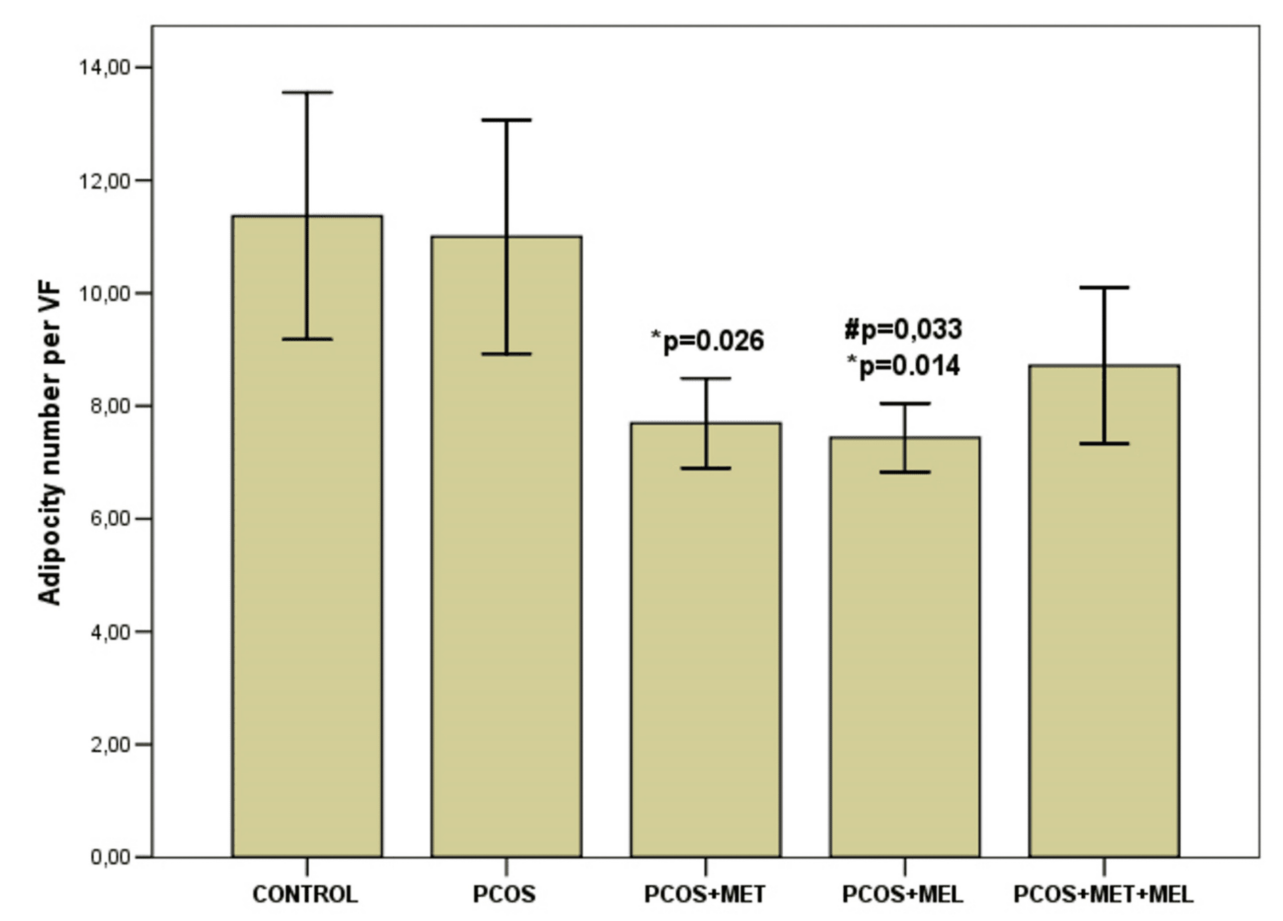
Adipocyte number per visual field across groups Results are mean ± standard deviation (x± SD),VF-visual field;  *significant to Control Group; ** significant to to PCOS; #significant to PCOS+MET

**Figure 6:**
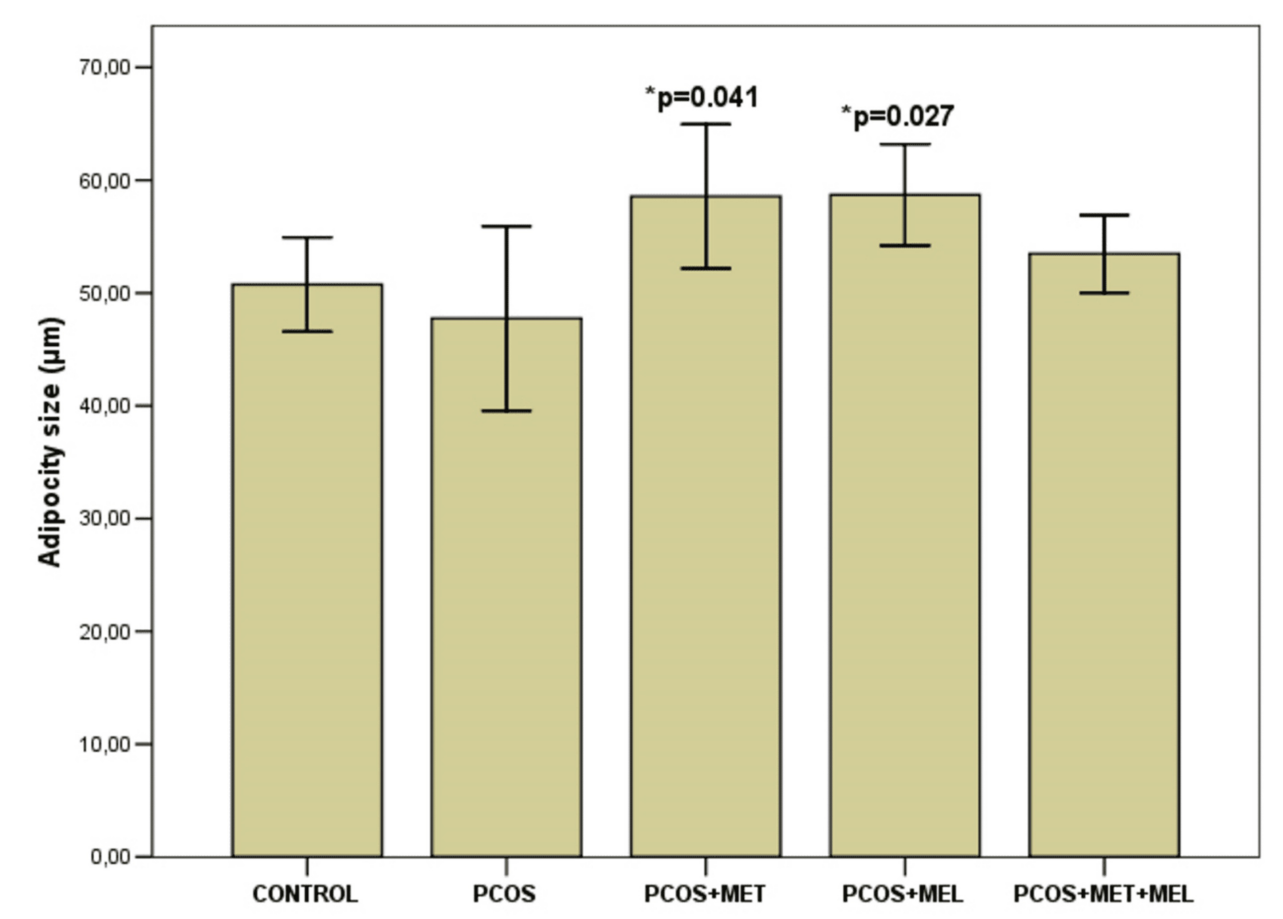
Adipocyte size among groups Results are mean ± standard deviation (x± SD), *significant to Control Group

**Figure 7:**
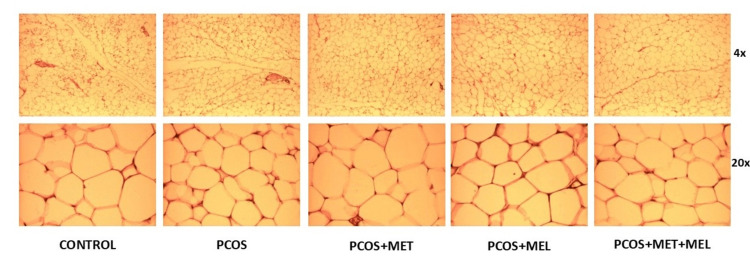
Representative photomicrographs of white adipose tissue - HE stain The control group showed a regular lobular organisation of unilocular adipocytes, mainly in white adipose tissue. PCOS rats had multilocular adipocytes with a prominent capillary network. The metformin-treated group had poorly defined lobules of predominantly unilocular, enlarged adipocytes with thin nuclei, and rarely brown tissue. The melatonin group has irregular lobules with heterogeneous adipocytes with flattened nuclei and occasional brown tissue. The Metformin+melatonin group had regularly arranged, uniform adipocytes in white tissue and few blood vessels.

While total visceral fat mass did not differ significantly across groups (p = 0.803), notable histological differences were observed (Table 3, Figures 5-7). The number of adipocytes per visual field was significantly reduced in both metformin- and melatonin-treated animals compared with controls (p = 0.026 and p = 0.014, respectively). A similar trend was evident between the PCOS model and the melatonin group (p = 0.033), but not in the metformin-only group. Interestingly, mean adipocyte size was smaller in the PCOS model than in either treatment group (p < 0.05). The melatonin + metformin group displayed relatively uniform unilocular adipocytes arranged in regular lobules with sparse vascularization, resembling control morphology (Figure 7). Ovarian analysis paralleled these findings: the PCOS model showed a pronounced theca interna thickening (22.18 ± 2.89 µm), which was significantly reduced following melatonin (13.72 ± 1.22 µm) or combination therapy (12.85 ± 1.06 µm; p < 0.001).

**Table 2:** Histological analysis results Comparison of values was performed by one-way analysis of variance (ANOVA), after which Bonferroni’s test for multiple comparisons was applied. Results are mean ± standard deviation (x± SD). VF-visual field; AFC-antral follicle count; ACP-antral follicle percentage. *significant to CG; ** significant to  PCOS; # significant to PCOS & metformin

Variable	CG	PCOS	PCOS+ metformin	PCOS+ melatonin	PCOS+ melatonin+ metformin	p
Adipose tissue (g)	3.93±2.14	3.16±0.48	3.51±1.26	3.44±0.86	3.13±0.80	0.803 F=0.405
Adipocyte number per VF	11.36±2.68	11.0±2.53	7.69±0.97 *p=0.026	7.43±0.74 *p=0.014 **p=0.033	8.71±1.70	0.002 F=0.5627
Adipocyte size (µm)	50.75±5.12	47.75±10.01	58.56±7.82 **p=0.048	58.70±5.50 **p=0.041	53.48±4.22	0.040 F=2.949
AFC per cut	5.0±2.75	7.0±4.28	4.66±1.6	5.16±2.13	6.83±2.85	0.495 F=0.872
ACP (%)	30.33±13.57	48.95±30.2	27.05±13.44	34.76±13.91	32.57±13.77	0.300 F=1.291
Theca interna (µm)	12.67±2.20	22.18±2.89 *p<0.001	18.18±2.40 * p=0.001 **p=0.027	13.72±1.22 **p<0.001 ^#^p=0.01	12.85±1.06 **p<0.001 ^#^p=0.002	<0.001 F=23.929

Correlation analysis

A statistically significant positive correlation was found between interleukin-1 beta (IL-1β) levels and the increase in abdominal circumference in the control group (rho = 0.956; p < 0.01) (Table 3).

**Table 3:** Correlation between inflammatory parameters and anthropometric variables in the control group Correlation was assessed using Pearson’s correlation coefficient (r) or Spearman’s rank correlation coefficient (rho), as appropriate. rho - Spearman’s rank correlation coefficient; r - Pearson’s correlation coefficient; ** p < 0.01; WG – weight gain; AC-abdominal circumference; TC – thoracic circumference; L- length

Variable	Interleukin -1β (pg/mL)	TNF α (pg/mL)	C-reactive protein (mg/L)
WG (g)	Rho= -0.429	r= 0.076	Rho= -0.655
AC (cm)	Rho= 0.956**	r= -0.100	Rho= 0.548
TC(cm)	Rho= -0.617	r= -0.260	Rho= -0.424
L(cm)	Rho= 0.696	r= 0.555	Rho= -0.266

A statistically significant positive correlation was found between TNF-alpha levels and body weight gain (rho = 0.927; p < 0.01) (Table 4), as well as a statistically significant negative correlation between C-reactive protein levels and an increase in body length (rho = -0.826; p < 0.05) in the PCOS model group.

**Table 4:** Correlation between inflammatory parameters and anthropometric variables in the PCOS model group Correlation was assessed using Pearson’s correlation coefficient (r) or Spearman’s rank correlation coefficient (rho), as appropriate. * - p<0.05; ** p < 0.01; WG – weight gain; AC-abdominal circumference; TC – thoracic circumference; L- length

Variable	Interleukin -1β (pg/mL)	TNF α (pg/mL)	C-reactive protein (mg/L)
WG (g)	Rho= 0.001	r= 0.927**	Rho= -0.282
AC (cm)	Rho= -0.137	r= 0.249	Rho= -0.219
TC(cm)	Rho= 0.454	r= 0.011	Rho= -0.433
L(cm)	Rho= 0.313	r= 0.338	Rho= -0.826*

In the PCOS model group, a significant positive correlation was found between TNF-alpha levels and the number of antral follicles per section (r = 0.844; p < 0.05) (Table 5).

**Table 5:** Correlation between inflammatory and histological parameters of the reproductive system in the PCOS model group Correlation was assessed using Pearson’s correlation coefficient (r) or Spearman’s rank correlation coefficient (rho), as appropriate. *p < 0.05; TFC-total follicle count; AFC-antral follicle count; ACP-antral follicle percentage

Varijabla	Interleukin -1β (pg/mL)	TNF α (pg/mL)	C-reactive protein (mg/L)
Theca interna (µm)	Rho= 0.134	r= 0.327	Rho= -0.715
TFC per cut	Rho= 0.118	r= 0.329	Rho= -0.423
AFC per cut	Rho= -0.058	r= 0.844*	Rho= -0.278
ACP (%)	Rho= -0.058	r= 0.409	Rho= -0.093

A statistically significant positive correlation was found between TNF-alpha levels and the increase in abdominal circumference (rho = 0.853; p < 0.05) in the PCOS + metformin + melatonin group (Table 6).

**Table 6:** Correlation between inflammatory parameters and anthropometric variables in the PCOS + metformin + melatonin group Correlation was assessed using Pearson’s correlation coefficient (r) or Spearman’s rank correlation coefficient (rho), as appropriate. * p < 0.05;  WG – weight gain; AC-abdominal circumference; TC – thoracic circumference; L- length

Variable	Interleukin -1β (pg/mL)	TNF α (pg/mL)	C-reactive protein (mg/L)
WG (g)	Rho= 0.334	r= 0.413	Rho= -0.139
AC (cm)	Rho= 0.116	r= 0.853*	Rho= 0.399
TC(cm)	Rho= 0.516	r= 0.397	Rho= 0.696
L(cm)	Rho= 0.638	r= 0.054	Rho= -0.399

## Discussion

The present study confirms that testosterone-induced PCOS in juvenile female rats reproduces the cardinal features of the syndrome - persistent estrus, increased abdominal circumference (AC), and characteristic ovarian histopathology - consistent with human PCOS [23,25]. While previous studies have demonstrated that prolonged testosterone exposure promotes visceral obesity [23,25], this phenomenon was not observed here. Total body weight and visceral fat mass remained comparable across groups, suggesting that androgen-induced PCOS in this model may trigger regional lipid redistribution rather than global adiposity.

Subcutaneous adipose tissue (SAT), which exhibits a higher intrinsic androgen production rate, may play an underrecognized role in sustaining PCOS-related hyperandrogenism. Inflammation within adipose depots disrupts lipid handling, increasing free fatty acid (FFA) efflux, which in turn activates pro-inflammatory pathways such as TLR4/NF-κB signaling in adipose and hepatic tissues, thereby amplifying systemic metabolic inflammation and insulin resistance [11]. In our model, although melatonin tended to reduce body weight, the difference did not reach statistical significance, consistent with previous findings [24]. It is possible that the exposure period was insufficient for measurable systemic weight differences, or that inter-batch variations in baseline body weight obscured subtle effects. Nonetheless, the observed abdominal enlargement, altered adipocyte morphology, and stable total fat mass suggest a shift in lipid compartmentalization - a process often preceding overt weight gain.

The thoracic-to-abdominal circumference ratio (TC/AC) was increased in the PCOS model, consistent with the findings of Pai et al. [24]. Both metformin and melatonin partially normalized these anthropometric indices, with the combination therapy showing the most pronounced effect. This supports previous observations that melatonin mitigates central adiposity and improves body composition in experimental PCOS models [24,27].

Cytokine analysis provides additional insight into melatonin's anti-inflammatory effects. While IL-1β levels were not markedly elevated in the PCOS model compared to controls, melatonin significantly reduced its concentration, particularly in combination with metformin. This is consistent with reports that melatonin supplementation suppresses IL-1 and TNF-α gene expression in PCOS models and clinical studies [27,28]. Interestingly, TNF-α levels remained unchanged in our testosterone-induced model, suggesting that androgen exposure alone may not be a strong inducer of systemic cytokine secretion but may instead drive oxidative stress-mediated pathways. The temporal kinetics of cytokine expression could also account for this discrepancy, as TNF-α typically peaks earlier than IL-1β in inflammatory cascades.

Histomorphological evaluation of the ovaries revealed multiple antral and atretic follicles, with rare corpora lutea, in PCOS rats, consistent with previous androgen-induction models [24,29]. Granulosa cell disorganization and thickening of the theca interna were evident, features that reflect hyperandrogenic stimulation and increased LH sensitivity [28]. Melatonin treatment led to a partial normalization of these alterations, with the combination of melatonin and metformin producing the most significant improvement - manifested by restoration of granulosa lamellarity and thinning of the theca layer. These findings suggest that melatonin's antioxidant and anti-inflammatory effects may help restore follicular maturation and ovulatory function when used alongside metformin.

Although the number of antral follicles showed a tendency toward an increase, the difference did not reach statistical significance, consistent with prior reports [24,30]. The combined data suggest that melatonin primarily exerts its benefit through metabolic and paracrine modulation rather than direct gonadal effects.

Taken together, this study provides evidence that melatonin, alone or in combination with metformin, can attenuate visceral adipose inflammation and improve ovarian histoarchitecture in androgen-induced PCOS. The observed decline in IL-1β and normalization of adipocyte morphology highlight its capacity to modulate both immune and metabolic compartments.

Certain limitations should be acknowledged. The relatively short treatment duration and lack of detailed hormonal or oxidative stress analyses may have constrained the detection of broader metabolic changes. Nonetheless, the consistency of histological and inflammatory findings supports the robustness of the observed effects.

## Conclusions

Prolonged testosterone exposure in prepubertal rats successfully reproduced hallmark features of PCOS, including disrupted estrous cyclicity, abdominal enlargement, and characteristic ovarian morphological changes. Although limited serum volume prevented comprehensive hormonal profiling, the model was confirmed by consistent clinical and histological indicators of hyperandrogenism. Melatonin demonstrated beneficial effects across several PCOS-related outcomes and, in some parameters, was more effective than metformin. Notably, improvements were observed in abdominal circumference, TC/AC ratio, inflammatory markers, and ovarian structure.

While combination therapy showed favorable trends in multiple outcomes, including reduced IL-1β levels and normalized ovarian histoarchitecture, its impact on adipocyte size and number did not consistently reach statistical significance. Therefore, conclusions regarding the synergistic effects of melatonin and metformin should be interpreted with caution. Future research should expand cytokine profiling, extend treatment duration, and include molecular analyses of adipose tissue to better understand the pathways underlying these effects and to confirm potential therapeutic synergy.
